# Performance measures of the specialty referral process: a systematic review of the literature

**DOI:** 10.1186/1472-6963-11-168

**Published:** 2011-07-13

**Authors:** James P Guevara, Diane Hsu, Christopher B Forrest

**Affiliations:** 1 PolicyLab: Center to Bridge Research, Practice, & Policy, The Children's Hospital of Philadelphia, Philadelphia, PA, USA; 2 Division of General Pediatrics, The Children's Hospital of Philadelphia, Philadelphia, PA, USA; 3 Center for Clinical Epidemiology and Biostatistics, University of Pennsylvania, Philadelphia, PA, USA; 4 Leonard Davis Institute of Health Economics, University of Pennsylvania, Philadelphia, PA, USA

**Keywords:** Primary Care, Specialists, Referral-Consultation, Coordination, Quality of Care

## Abstract

**Background:**

Performance of specialty referrals is coming under scrutiny, but a lack of identifiable measures impedes measurement efforts. The objective of this study was to systematically review the literature to identify published measures that assess specialty referrals.

**Methods:**

We performed a systematic review of the literature for measures of specialty referral. Searches were made of MEDLINE and HealthSTAR databases, references of eligible papers, and citations provided by content experts. Measures were eligible if they were published from January 1973 to June 2009, reported on validity and/or reliability of the measure, and were applicable to Organization for Economic Cooperation and Development healthcare systems. We classified measures according to a conceptual framework, which underwent content validation with an expert panel.

**Results:**

We identified 2,964 potentially eligible papers. After abstract and full-text review, we selected 214 papers containing 244 measures. Most measures were applied in adults (57%), assessed structural elements of the referral process (60%), and collected data via survey (62%). Measures were classified into non-mutually exclusive domains: need for specialty care (N = 14), referral initiation (N = 73), entry into specialty care (N = 53), coordination (N = 60), referral type (N = 3), clinical tasks (N = 19), resource use (N = 13), quality (N = 57), and outcomes (N = 9).

**Conclusions:**

Published measures are available to assess the specialty referral process, although some domains are limited. Because many of these measures have been not been extensively validated in general populations, assess limited aspects of the referral process, and require new data collection, their applicability and preference in assessment of the specialty referral process is needed.

## Background

Access to specialty care in developed countries occurs mainly through primary care referrals [1]. Specialists provide important services related to resolution of clinical uncertainty, provision of long-term medical therapy for patients with unusual or complex medical problems, and provision of specialized technologies [2,3]. Nonetheless, a clear consensus on the availability and role of specialists is lacking, which likely contributes to the marked variation in use of specialty care across regions and countries [4].

The optimal availability of specialty care has been a long-standing matter of contentious debate [5]. Workforce models that extrapolate current levels of specialist utilization while accounting for demographic trends project an impending shortage, particularly in the U.S [6]. These models assume that current demand for specialists per population will remain constant or even grow over time [7]. Others have argued that more appropriate use of the skills and expertise of specialists would bring supply and demand into balance within the constraints of future workforce supply [8,9]. For example, some large integrated health systems use fewer specialists per 1,000 members than conventional open-access systems and achieve comparable outcomes [10-12].

While consensus on the role of primary care clinicians in specialty referrals has been established, similar consensus on the normative role of specialists has not been achieved [4]. For example, primary care clinicians initiate referrals and coordinate specialty care when diagnostic uncertainty arises or treatment becomes complex [13,14]. Responsibilities for primary care clinicians in this role include ensuring that the health needs of patients are met, services are integrated across providers and over time, and patients are linked with relevant community resources [15-17]. The benefits of these primary care tasks have been established empirically [13,18].

Empirical research on the role of specialists in the referral process is less well defined and mainly involves comparisons of primary care vis-à-vis specialist care without strong methodological rigor [19]. Overall, the literature suggests that care provided by specialists compared with that provided by generalists is more costly due to the addition of expensive tests and highly selective treatments [20-22]. In addition, care provided by specialists is more likely to be evidence based within the specialist's area of expertise but may be less evidence-based and associated with poorer outcomes outside their expertise. Several studies have examined care provided jointly by primary care clinicians and specialists for patients with chronic disease and have found that it is associated with better outcomes in comparison with either acting alone [23-26].

With health care costs rising, efforts to control costs and improve the efficiency of the specialty referral process have gained renewed traction in current healthcare debates [27-29]. Measures of the specialty referral process are needed to inform the ongoing debate on the availability and appropriate role of specialists in the healthcare delivery system. To address this need, we sought to identify published measures that assess the performance of the specialty care process including the interface between primary care and specialty care. Such measures will be of interest to healthcare organizations to allow them to benchmark current practices, assist providers in meeting standards of care, and determine the most rational ways to organize the primary-specialty care interface. Such measures will also be of interest to academic researchers who seek to better understand the complexities of the current referral process and to develop and test innovative improvements in specialty referrals. Therefore, the aims of the study were to 1) identify published measures of the specialty referral process through a systematic review of the literature, 2) categorize the measures according to a conceptual framework of the specialty referral process, and 3) assess the content validity of identified metrics, the conceptual framework developed, and the assignment of metrics to given domains of the framework among a group of specialty referral content experts.

## Methods

### Conceptual Framework

**Figure 1:**
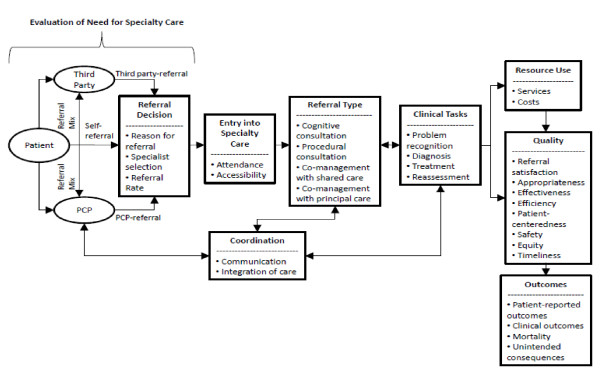
**Specialty Referral Process**. Referral process is a series of health-related events beginning when a patient has an assessment of need for specialty care, progresses to the decision to make a referral and enter into specialty care, and ends with the completion of referred care. The process encompasses communication and coordination between referring and consultant clinicians regarding tasks to be completed. The overall referral process results in a given resource utilization, quality, and outcomes for a patient.

We developed a conceptual framework to guide the identification and categorization of measures of the specialty referral process (Figure 1). This framework was developed from a review of the literature on specialty referrals and conceptual frameworks and underwent several iterations based on suggestions from a panel of 10 content experts [4,30-32]. According to the framework, the specialty referral process represents a series of health-related events or stages (i.e. domains) to diagnose and/or manage a health condition. In the first stage, a patient considers whether to seek care for a health problem from a specialist (Evaluation of Need for Specialty Care). Consultation regarding the referral decision may be made with a referral source such as a primary care clinician or emergency medicine clinician. Once a decision is made to seek a referral, the initiation of a referral involves the reason(s) for referral, urgency of referral, and selection of specific specialty (Referral Initiation). In the next stage, a patient makes an appointment with a specialist (Entry into Specialty Care), which can be delayed or hindered by a number of access-related barriers including geographic, organizational, and financial factors. Once a patient visit is completed, a specialist may direct a number of diagnostic or therapeutic procedures, consider duration of follow-up, and suggest additional referrals (Clinical Tasks). The extent of the specialist involvement may be short-term and involve consultation for a diagnosis or specified procedure or may be long-term and involve co-managed care or transferred care (Referral Type). Information regarding clinical tasks and their outcomes can be exchanged between the specialist and referring clinician, and care can be distributed between the specialist and the referring clinician (Coordination). The clinical tasks involved in the referral process will consume an amount and monetary value of health services (Resource Use) and reflect a degree of quality consistent with best available evidence based on the patient's and provider's expectations of the referral (Quality). The referral process from initiation to completion may impact on the patient's health problem and his or her overall health and quality of life (Outcomes).

### Search Strategy

We conducted a systematic review of the published literature to identify relevant papers that contain measures of the specialty referral process. We searched the electronic databases MEDLINE and HealthSTAR from January 1973 to June 2009, to coincide with the emergence of managed care papers in the published literature to the present. We used a search strategy intended to be highly sensitive for identification of specialty referral articles from a previous study [4]:

(("Primary health care"[MeSH Terms] OR generalists[Text Word]) AND ("specialism"[MeSH Terms] OR special*[Text Word] OR subspecial*[Text Word])) AND ((coordination[Text Word] OR communication[Text Word] OR shared decision making[Text Word] OR co-management[Text Word] OR shared care[Text Word] OR integrated care[Text Word] OR multidisciplinary care[Text Word]) OR "referral and consultation"[MeSH Terms]))

The reference lists of all eligible papers were reviewed for additional eligible papers. In addition, published papers nominated by content experts were reviewed for eligibility. This research was granted an exemption from review by the Institutional Review Board at the Childrens Hospital of Philadelphia.

### Eligibility Criteria

Studies were eligible if they were (1) published in peer-reviewed journals or government reports in 1973 or later, (2) performed in one of the member Organization for Economic Cooperation and Development (OECD) countries or Israel, (3) contained an operational measure(s) of the referral process with prior or current data supporting the measure's validity (content, concurrent, or construct) or reliability (inter-rater, test-retest, or internal consistency), and (4) addressed referrals to a recognized medical specialty. Operational measures included instruments, questionnaires, and other tools that purport to measure a component of the specialty referral framework. Measures did not have to be specific to referrals but could report on the outcomes or quality of referrals. We accepted articles that demonstrated validity or reliability of the metric within the presentation of results of the paper (e.g., a metric that changes in hypothesized directions with outcome variables) or made reference to a prior publication that reported on the measure's validity or reliability.

A variety of exclusion criteria were employed to increase the specificity of article selection. First, we excluded studies that employed interventions which defined the duration and type of specialist involvement; surveys in which general attitudes of the referral process were the focus; and, studies of hypothetical referral scenarios, because we wanted to capture measures involved in actual referral behavior. Second, we excluded review papers, although we utilized these to identify papers containing referral metrics. Third, we excluded studies involving only referrals to clinicians not part of the American Board of Medical Specialties (e.g. physical therapists and dentists), since these may not involve clinician-to-clinician communication and coordination of referrals. We did capture referrals to psychologists, as they were often viewed along with psychiatrists as comprising mental health referrals in the literature. Fourth, we excluded referrals to hospital-based specialists including emergency departments, anesthesiologists, radiologists, and inpatient units of hospitals due to the urgent nature of many of these referrals. Fifth, we excluded curbside consultations, since data on these consultations are difficult to capture.

The title and abstract of all identified papers were reviewed for potential eligibility. The full-text of each potentially eligible paper was then reviewed for eligibility independently by three investigators (JG, DH, CF), two of which were assigned to each paper. Differences between investigators were settled by consensus of all investigators as to whether the pre-specified inclusion and exclusion criteria were met. After a training period of approximately 50 papers, we had a final overall agreement rate of 87% with κ = 0.60 (p < 0.001).

Eligible papers were abstracted using standardized abstraction forms that had been piloted prior to study initiation. Since papers may contain more than one relevant referral measure, we abstracted information on all metrics contained in each eligible paper that met eligibility criteria. We abstracted information on each metric's definition as given in the published paper, its components and formula for calculation (numerator, denominator), data type (integer, ratio, proportion), data source (administrative data, surveys, chart abstraction), validity measure, reliability measure, and use in the paper. We categorized each metric according to the Donabedian framework of structure, process, and outcome [33]. We also abstracted information from each paper on the specific specialty involved, the patient population (children, adults), and disease state involved if any. Eligible metrics were then categorized into one or more domains and sub-domains of the conceptual framework of the specialty referral process (Figure 1).

To clarify measures and obtain missing information, we contacted corresponding authors of eligible papers to provide explanations on how metrics were constructed, how they were validated, what their purpose was, and to obtain a copy of the instruments if available. Metrics were linked to all source studies, defined as the first published study containing an identified metric. Subsequent studies that contained a given metric were also linked to source studies.

### Expert Panel

We identified a pool of content experts on specialty referrals through our literature search and by nomination of other experts. We selected a pool of 10 who agreed to assess the content validity of the metric set and the corresponding conceptual framework and to propose additional papers not previously identified in our literature search. The expert panel included health services researchers, clinical administrators, insurance executives, and practicing physicians from the U.S. and the U.K. Names of panel members can be found in the acknowledgement section.

## Results

**Figure 2:**
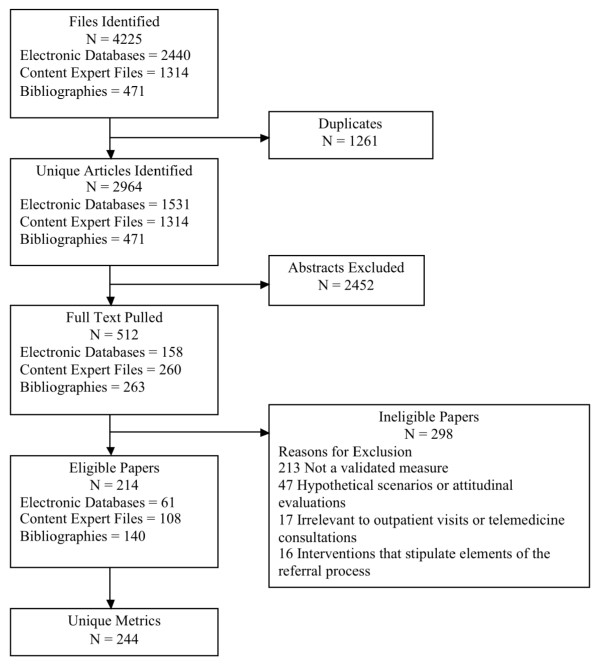
**Flow chart of Literature Search**.

We identified 4,225 studies from our search of MEDLINE and HealthSTAR databases supplemented by additional papers from content experts and searches of the reference lists of eligible papers (Figure 2). After accounting for 1,261 duplicate articles, we identified 2,964 unique articles and government reports. Based on review of abstracts, we excluded 2,452 studies based on exclusion criteria and pulled the full text of 512 papers for a more comprehensive review. After reviewing the full-text of these potentially eligible papers, we excluded an additional 298 articles, which left 214 eligible papers containing 244 unique referral metrics (Additional File 1). We attempted to contact 126 corresponding authors, 24 of whom had no valid contact information. A majority of the remaining authors (90 of 102, 88%) responded to our inquiries and provided us with additional information or the full instruments if available. Based on this correspondence, we were able to obtain additional information for 129 metrics that was not readily apparent in the source studies' methodologies. Of these, 79 metrics (61%) were available in online resources, were provided by corresponding authors, or were embedded in the full text of papers.

A majority of the eligible metrics (57%) were assessed exclusively in adult patient populations, while a quarter (26%) of the metrics were assessed exclusively in children and the remainder (17%) were assessed in both child and adult populations. The majority of the measures (63%) examined referrals to medical specialties, as opposed to surgical and mental health specialties. The most common individual subspecialty reported in the studies was mental health (42%), followed by otolaryngology (30%), dermatology (28%), neurology (23%), general surgery (23%), cardiovascular medicine (22%), and ophthalmology (21%) or orthopedic surgery (21%). A number of measures (27%) were evaluated in multiple subspecialties.

With consultation from the expert panel, measures were categorized into domains and sub-domains of the conceptual framework (Table 1). The majority of measures were from one of four domains: Referral Initiation (73 metrics, 30%), Entry into Specialty Care (53 metrics, 22%), Coordination (60 metrics, 26%), and Quality (57 metrics, 23%). Referral Initiation metrics included mainly measures of the reason for referral and selection of specialist (27 metrics) and rates of referral (42 metrics). Entry into Specialty Care metrics were comprised of measures of accessibility (46 metrics) with few assessing attendance at the specialist visit. Coordination metrics consisted of measures of communication of the reason and expectations for referral to the specialist (48 metrics), with few assessing integration of care with referring clinicians (17 metrics). Quality metrics were mainly comprised of timeliness of initial specialty visits (23 metrics) and satisfaction (21 metrics). There were no identified quality measures assessing the safety or equity of referrals and few assessing the effectiveness, efficiency, or patient-centeredness of referrals. There were few measures that comprised Need for Specialty Care (14 metrics, 6%), Clinical Tasks (19 metrics, 8%), Referral Type (3 metrics), Resource Use (13 metrics, 5%), or Outcomes (9 metrics, 4%).

**Table 1:** Metric Domains, Sub-domains, and Exemplary Metrics

Domain	Sub-domains	# Metrics	Metric Example
Evaluation of Need for Specialty Care (n = 14)	Referral Source	14	Proportion of self-referred visits [38]
	Referral Decision	0	
Referral Initiation (n = 73)	Reason for referral	4	Proportion of referrals for advice on diagnosis and treatment [3]
	Specialist selection	27	Proportion of PCPs who referred at patient request [39]
	Referral rate	42	# Referrals to a specialist per 100 PCP visits [40]
			
Entry into Specialty Care (n = 53)	Attendance	7	Proportion of referred patients who attended first specialty visit [41]
	Accessibility	46	Total # providers per 100,000 people per state [42]
Coordination (n = 60)	Communication	48	Proportion of specialist reports by letter or e-mail [43]
	Integration of care	17	Proportion of PCPs who received feedback from a specialist [43]
Referral Type (n = 3)	Consultation	3	Proportion of referrals for procedural consultation [44]
	Co-management	3	Proportion of referrals for co-management with shared care [44]
			
Clinical Tasks (n = 19)	Problem recognition	10	Proportion of letters including a patient's condition [45]
	Diagnosis	18	Proportion of letters including a patient's diagnosis [45]
	Treatment	17	Proportion of letters including treatment recommendation [45]
	Reassessment	12	Proportion of letters including follow-up arrangements [45]
Resource Use (n = 13)	Services	12	Primary and specialty visits per patient per 30 days [46]
	Costs	13	Total annual specialty expenditures [47]
Quality (n = 57)	Appropriateness	13	Proportion of patients who received an unnecessary referral [48]
	Effectiveness	4	Proportion of PCPs who adhered to a specialists' recommendations [49]
	Efficiency	1	Proportion of PCPs who believed a specialist was minimizing costs [50]
	Equity	0	
	Patient-centeredness	8	Proportion of patients who thought that specialty care was helpful[51]
	Referral satisfaction	21	Proportion of patients who were satisfied with specialty care [52]
	Safety	0	
	Timeliness	23	Average length of time spent for a specialty visit [53]
Outcomes (n = 9)	Health status	3	Change in depression score on Hopkins Symptom Checklist [54]
	Mortality	2	Proportion of surviving patients per year [55]
	Functional status	3	Change in score on Pain Interference Scale [56]
	Unintended Consequences	0	

A majority of eligible metrics (60%) assessed structural features of referrals (Table 2), while a minority assessed processes (34%) or outcomes (19%). Most metrics (98%) included a measure of validity with the most common type being construct validity (88%), followed by content validity (42%). Few measures included criterion validity (7%) or an assessment of reliability (16%).

**Table 2:** Characteristics of Referral Metrics by Domain

Characteristic	Need^1 ^ N = 14	Initiation^2 ^ N = 73	Entry^3 ^ N = 53	Coordination N = 60	Type^4 ^ N = 3	Tasks^5 ^ N = 19	Resources^6 ^ N = 13	Quality N = 57	Outcomes N = 9	All Domains N = 244
Donabedian Domains n(%)	14(100)	73(100)	53(100)	0(0)	0(0)	0(0)	0(0)	0(0)	0(0)	147(60.2)
Structure	0(0)	0(0)	0(0)	60(100)	3(100)	19(100)	13(100)	0(0)	0(0)	84(34.4)
Process	0(0)	0(0)	0(0)	0(0)	0(0)	0(0)	0(0)	57(100)	9(100)	46(18.9)
Outcome	14(100)	70(95.9)	52(98.1)	59(98.3)	3(100)	19(100)	12(92.3)	57(100)	9(100)	239(98.0)
Validity, n(%)										
Content	2(14.3)	22(30.1)	24(45.3)	53(88.3)	2(66.7)	12(63.2)	1(7.7)	25(43.9)	3(33.3)	103(42.2)
Criterion	1(7.1)	5(6.8)	4(7.5)	1(1.6)	0(0)	3(15.8)	2(15.4)	4(7.0)	0(0)	17(7.0)
Construct	14(100)	67(91.8)	49(92.5)	40(58.6)	3(100)	14(73.7)	10(76.9)	54(94.7)	7(77.7)	215(88.1)
Reliability, n(%)	0(0)	15(20.5)	7(10.1)	17(27)	0(0)	4(21.1)	1(7.7)	10(17.5)	1(11.1)	38(15.6)
Inter-Rater	0(0)	2(2.7)	2(2.9)	7(11.1)	0(0)	1(5.3)	0(0)	3(7.9)	0(0)	12(4.9)
Test-Retest	0(0)	1(1.4)	0(0)	3(4.8)	0(0)	1(5.3)	0(0)	0(0)	0(0)	4(1.6)
Internal Consistency	0(0)	12(16.4)	5(7.2)	13(20.6)	0(0)	3(15.8)	1(7.7)	8(21.1)	1(11.1)	28(11.5)
Data Source, n(%)										
Questionnaire	6(42.9)	51(69.9)	30(56.6)	46(76.7)	3(100)	11(57.9)	2(15.4)	42(73.7)	6(66.7)	152(62.3)
Administrative	4(28.6)	8(11.0)	24(45.2)	1(1.6)	0(0)	2(10.5)	10(76.9)	4(7.0)	1(11.1)	49(20.1)
Chart Review	2(14.3)	25 (34.2)	9(17.0)	22(34.9)	0(0)	10(52.6)	1(7.7)	18(31.6)	2(22.2)	75(30.7)
Not reported	2(14.3)	0(0)	0(0)	0(0)	0(0)	0(0)	0(0)	0(0)	0(0)	2(0.8)

^1^'Need' refers to a patient's evaluation of need for specialty care

^2^'Initiation' refers to the steps taken to refer a patient

^3^'Entry' refers to a patient's entry into specialty care

^4^'Type' refers to the relationship between a PCP and referred specialist

^5^'Tasks' refers to the clinical tasks performed during care

^6^'Resources' refers to the usage and costs of services

Eligible measures were derived from several different data sources, which were grouped into one of the following three categories: questionnaire, administrative data, or chart review. To improve the accuracy and precision of data, researchers utilized several data sources in collecting information for an individual metric, so a single operational measure may not be mutually exclusive to a particular type of data. Questionnaires included self-administered and staff-aided instruments completed by the patient, primary care physician, or specialist, and a majority of the measures (62%) were derived from this source of information (Table 2). Approximately one fifth of the metrics (20%) utilized administrative data, which included information readily available in health plans, public databases, scheduling and billing databases, and physician claims files. Roughly one third (31%) of the measures were derived from chart reviews, which included referral letters, paper and electronic medical records, and referral logs maintained by office managers. Very few metrics (1%) in this review did not report on data sources.

## Discussion

In this review, we found that available measures were limited in assessing the process and outcomes of referrals. For example, we identified few measures that assess the adequacy and appropriateness of coordination of referrals, an important process measure intended to capture the allocation of tasks and assignment of roles among primary care and specialty providers in coordinating care for patients. In addition, we identified few measures that assess the quality, resource consumption, and outcomes of referrals. Despite the availability of a number of validated measures designed to capture health services use or patient outcomes and well-being, few of these measures have been utilized in studies of specialty referrals [34-37]. Process measures help to tie structures to their intended outcomes. Clearly, measures that are intended to assess the process of referrals like care coordination and outcome measures are needed in order to better evaluate current referral quality.

The majority of measures we identified in this study require collection of new data through either questionnaires or chart reviews. Few (20%) are designed to rely on existing administrative data. This presents challenges in the ability of metrics to be used to compare referral data across provider groups and health plans. However, with the national movement toward electronic health records (EHR) in the U.S. and elsewhere, it may be conceivable to embed key metrics within the EHR and collect referral data for cross-system comparisons. This will require consensus on the selection of key metrics among the many that are available.

This study has limitations that should be addressed. First, as with all systematic reviews, there exists the possibility of publication bias in which we missed important specialty care measures. We took a number of steps to limit this bias by utilizing a previously validated search strategy for identifying papers on specialty referrals, a thorough review of the reference lists of all eligible papers, and contact with experts in the field who could nominate additional papers to supplement our list. Second, our review identified a large pool of available validated measures without providing an endorsement for a core set of measures. Future study is needed to identify a subset of these measures that can be endorsed for more general use.

## Conclusions

Operational measures of the specialty referral process are available in the published literature. A majority of identified metrics were studied among adult patients and evaluated the frequency and reason for referrals, specialty accessibility and timeliness, communication, and satisfaction. In addition, a majority of the measures focused on structural components of the referral process; few measures assessed the process or outcomes of referrals. While measures in this review nearly universally included assessments of validity, few reported on reliability. Most measures relied on collection of new data rather than on existing administrative or claims data.

We believe that our study has important research implications. First, a set of referral measures such as those identified in this study can be utilized to assess the performance of the current referral system and to evaluate the comparative effectiveness of interventions to improve the quality and efficiency of specialty referrals. A toolkit is available on-line at http://www.research.chop.edu/tools/psrt/index.php which lists searchable characteristics of these measures and provides their link to the conceptual framework. Identification and categorization of published measures of specialty care referral can be considered the first step toward assessment of the referral system. Consensus on a core set of measures derived from this study can then be developed to uniformly measure specialty referrals and benchmark referral practices. Second, given the limited number of measures in key domains, researchers should target the development of new measures to fill these gaps and better assess the processes and outcomes of referrals. Third, the dynamics between primary and specialty clinicians are evolving as technological advancements become an important part of medical practices and electronic records, and electronic communication is changing the way information is managed and transferred. Finding ways to incorporate measures of specialty care and other aspects of health care delivery into electronic information systems can facilitate evaluation of current practices and contribute to the redesign of future specialty care referral processes.

## Competing interests

The authors declare that they have no competing interests.

## Authors' contributions

JG conceived of the study, wrote the protocol, participated in the data collection, conducted phone interviews with expert panel members, drafted and revised all drafts of the manuscript, and approved the final version. DH conducted the systematic review, abstracted eligible studies, helped conduct phone interviews with expert panel members, assisted in the drafting of the manuscript, and approved the final version. CF helped conceive of the study, participated in phone interviews with expert panel members, assisted in the drafting of the manuscript, and approved the final version.

## Authors' information

James P. Guevara is at the Perelman School of Medicine at the University of Pennsylvania and The Children's Hospital of Philadelphia.

Diane Hsu is at The Children's Hospital of Philadelphia.

Christopher B. Forrest is at the Perelman School of Medicine at the University of Pennsylvania and The Children's Hospital of Philadelphia.

## Pre-publication history

The pre-publication history for this paper can be accessed here:

http://www.biomedcentral.com/1472-6963/11/168/prepub

## Supplementary Material

Additional file 1 **Referral Appendix**. Reference list of all eligible papers from the Systematic Review Click here for file
