# Supplementary material for: Performance measures of the specialty referral process: a systematic review of the literature
Source: BMC Health Serv Res. 2011 Jul 13;11:168. doi: 10.1186/1472-6963-11-168 (PMC3155905; doi:10.1186/1472-6963-11-168)
Supplement: Additional file 1 — Referral Appendix. Reference list of all eligible papers from the Systematic Review [file 1472-6963-11-168-S1.DOC]

Appendix

Allison JJ, K. C., Cook EF, Gerrity MS, Orav EJ, Centor R. 1998. “The association of physician attitudes about uncertainty and risk taking with resource use in a Medicare HMO.” Medical Decision Making 18(3): 320-9.

Armstrong, D., J. Fry, and P. Armstrong. 1991. “Doctors' perceptions of pressure from patients for referral.” British Medical Journal 302(6786): 1186-88.

Armstrong D, B. N., Grace J. 1988. “Measuring General Practitioner Referrals: Patient, Workload, and List Size Effects.” The Journal of the Royal College of General Practitioners 38: 494-97.

Armstrong D, G. G. 1987. “Patterns of work in general practice in the Bromley health district.” Journal of the Royal College of General Practice 37(299): 264-6.

Asplin BR, R. K., Levy H, Lurie N, Crain AL, Carlin BP, Kellermann AL. 2005. “Insurance status and access to urgent ambulatory care follow-up appointments.” Journal Of the American Medical Association 294(10): 1348-54.

Attkisson CC, Z. R. 1982. “The client satisfaction questionnaire. Psychometric properties and correlations with service utilization and psychotherapy outcome.” Evaluation and Program Planning 5(3): 233-7.

Bachman, K. H. and D. K. Freeborn. 1999. “HMO physicians' use of referrals.” Social Science & Medicine 48(4): 547-57.

Baldwin LM, P. M., Larson EH, Lishner DM, Mauksch LB, Katon WJ, Walker E, Hart LG. 2006. “Modeling the mental health workforce in Washington State: using state licensing data to examine provider supply in rural and urban areas.” Journal of Rural Health 22(1): 50-8.

Basarab, T., S. E. Munn, and R. R. Jones. 1996. “Diagnostic accuracy and appropriateness of general practitioner referrals to a dermatology out-patient clinic.” British Journal of Dermatology 135(1): 70-73.

Beltramini, R. F. and A. K. Sirsi. 1992. “Informational influences on physician referrals.” Journal of Hospital Marketing 6(2): 101-26.

Bergmo, T. S. 1997. “An economic analysis of teleconsultation in otorhinolaryngology.” Journal of Telemedicine and Telecare 3(4): 194-99.

Berta, W., J. Barnsley, J. Bloom, R. Cockerill, D. Davis, L. Jaakkimainen, A. M. Mior, Y. Talbot, and E. Vayda. 2009. “Enhancing continuity of information: essential components of consultation reports.” Canadian Family Physician 55(6): 624-5 e1-5.

Bickell, N. A., K. S. Pieper, K. L. Lee, D. B. Mark, D. D. Glower, D. B. Pryor, and R. M. Califf. 1992. “Referral patterns for coronary artery disease treatment: gender bias or good clinical judgment?” Annals of Internal Medicine 116(10): 791-97.

Bird, J. C., G. J. Beynon, A. T. Prevost, and D. M. Baguley. 1998. “An analysis of referral patterns for dizziness in the primary care setting.” British Journal of General Practice 48(437): 1828-32.

Borowsky SJ, R. L., Skootsky SA, Shapiro MF. 1997. “Referrals by general internists and internal medicine trainees in an academic medicine practice.” The American journal of managed care 3(11): 1679-87.

Bourguet C, G. V., McCord G. 1998. “The consultation and referral process. A report from NEON. Northeastern Ohio Network Research Group.” The Journal of Family Practice 46(1): 47-53.

Bowling, A., K. Stramer, E. Dickinson, J. Windsor, and M. Bond. 1997. “Evaluation of specialists' outreach clinics in general practice in England: process and acceptability to patients, specialists, and general practitioners.” Journal of Epidemiology & Community Health 51(1): 52-61.

Bowling A, R. J. 2000. “The process of outpatient referral and care: the experiences and views of patients, their general practitioners, and specialists.” British Journal of General Practice 50(451): 116-20.

Bowling A, W. J. 1997. “Discriminative power of the health status questionnaire 12 in relation to age, sex, and longstanding illness: findings from a survey of households in Great Britain.” Journal of Epidemiology & Community Health 51(5): 564-73.

Boyle, P. J., H. Kudlac, and A. J. Williams. 1996. “Geographical variation in the referral of patients with chronic end stage renal failure for renal replacement therapy.” The Quarterly Journal of Medicine 89(2): 151-57.

Brock, C. 1977. “Consultation and referral patterns of family physicians.” The Journal of Family Practice 4: 1129-37.

Brunner-La Rocca HP, C. J., Kiowsk W. 2006. “Compliance by referring physicians with recommendations on heart failure therapy from a tertiary center.” Journal of Cardiovascular Pharmacology and Therapeutics 11(1): 85-92.

Byrd, J. C. and M. A. Moskowitz. 1987. “Outpatient consultation: interaction between the general internist and the specialist.” Journal of General Internal Medicine 2: 93-98.

Calman, N. S., R. B. Hyman, and W. Licht. 1992. “Variability in consultation rates and practitioner level of diagnostic certainty [see comments].” The Journal of Family Practice 35: 31-38.

Carpenter PJ, M. G., Del Gaudio AC, Ritzler BA. 1981. “Who keeps the first outpatient appointment?” The American Journal of Psychiatry 138(1): 102-5.

Cassady CE, S. B., Hurtado MP, Berk RA, Nanda JP, Friedenberg LA. 2000. “Measuring consumer experiences with primary care.” Pediatrics 105(4 Pt 2): 998-1003.

Chang RK, H. N. 1997. “Geographic distribution of pediatricians in the United States: an analysis of the fifty states and Washington, DC.” Pediatrics 100(2 Pt 1): 172-9.

Christensen, B., H. T. Sorensen, and C. E. Mabeck. 1989. “Differences in referral rates from general practice.” Family Practice 6: 19-22.

Clancy CM, F. P. 1997. “Utilization of specialty and primary care: the impact of HMO insurance and patient-related factors. .” The Journal of Family Practice 45(6): 500-8.

Committee, G. M. E. N. A. 1980. “Report of the Graduate Medical Education National Advisory Committee, Geographic Distribution Technical Panel.” Washington, DC: Government Printing Office.

Cook, N. L., J. Z. Ayanian, E. J. Orav, and L. S. Hicks. 2009. “Differences in specialist consultations for cardiovascular disease by race, ethnicity, gender, insurance status, and site of primary care.” Circulation 119(18): 2463-70.

Cook NL, H. L., O'Malley AJ, Keegan T, Guadagnoli E, Landon BE. 2007. “Access to specialty care and medical services in community health centers.” Health Affairs (Millwood) 26(5): 1459-68.

Cornwall, P. 1993. “Communication between general practitioners and child psychiatrists.” British Medical Journal 306(6879): 629-3.

Coulter, A., A. Noone, and M. Goldacre. 1989. “General practitioners' referrals to specialist outpatient clinics. I. Why general practitioners refer patients to specialist outpatient clinics [see comments].” British Medical Journal 299(6694): 304-06.

Coulter A, B. J., Agass M, Martin-Bates C, Tulloch A. 1991. “Outcomes of referrals to gynaecology outpatient clinics for menstrual problems: an audit of general practice records.” British Journal of Obstetrics and Gynaecology 98(8): 789-96.

Coulter A, B. J. 1993. “Effect of NHS reforms on general practitioners' referral patterns.” British Medical Journal 306(6875): 443-7.

Coulter A, N. A., Goldacre M. 1989. “General practitioners' referrals to specialist outpatient clinics. II. Locations of specialist outpatient clinics to which general practitioners refer patients.” British Medical Journal 299(6694): 306-8.

Coulter A, S. V., McPherson K. 1990. “Relation between general practices' outpatient referral rates and rates of elective admission to hospital.” British Medical Journal 301: 273-76.

Council on Graduate Medical Education, T. R. 1998. “Physician Distribution and Health Care Challenges in Rural and Inner-City Areas.” H. R. a. S. U.S. Department of Health and Human Services and Administration. Washington, DC.

Cowen, M. E. and M. W. Zodet. 1999. “Methods for analyzing referral patterns.” Journal of General Internal Medicine 14(8): 474-80.

Crombie DL, F. D. 1988. “General practitioner referrals to hospital: the financial implications of variability.” Health Trends 20(2): 53-6.

Crossley GM, H. A., Newble D, Jolly B, Davies HA. 2001. “Sheffield Assessment Instrument for Letters (SAIL): performance assessment using outpatient letters.” Medical Education 35(12): 1115-24.

Crump WJ, M. P. 1988. “Outpatient consultations from a family practice residency program: nine years' experience.” The Journal of the American Board of Family Practice 1(3): 164-6.

Cummingham, P. 2009. “Beyond parity: primary care physicians' perspectives on access to mental health care.” Health Affairs (Millwood) 28(3): 490-501.

Cummins RO, J. B., White PM. 1981. “Do general practitioners have different "referral thresholds"?” British Medical Journal (Clinical Research Edition) 282(6269): 1037-9.

Dale, J., J. Green, F. Reid, E. Glucksman, and R. Higgs. 1995. “Primary care in the accident and emergency department: II. Comparison of general practitioners and hospital doctors.” British Medical Journal 311(7002): 427-30.

Del Gaudio, A. C., P. J. Carpenter, L. S. Stein, and G. Morrow. 1977. “Characteristics of patients completing referrals from an emergency department to a psychiatric outpatient clinic.” Comprehensive Psychiatry 18(3): 301-07.

Dial TH, B. C., Haviland MG, Pincus HA. 1998. “Psychiatrist and nonphysician mental health provider staffing levels in health maintenance organizations.” The American Journal of Psychiatry 155(3): 405-8.

Dial TH, P. S., Bergsten C, Gabel JR, Weiner J. 1995. “Clinical staffing in staff- and group-model HMOs.” Health Affairs (Millwood) 14(2): 168-80.

Dickey, W. and J. I. Morrow. 1991. “Can outpatient non-attendance be predicted from the referral letter? An audit of default at neurology clinics.” Journal of the Royal Society of Medicine 84(11): 662-63.

Donaldson N, C. H., Green R. 2000. “Quality of information on hospice referral.” British Journal of General Practice 50(452): 219-20.

Donohoe MT, K. R., Wheeler DB, Chandra R, Chen A, Humphries N. 1999. “Reasons for outpatient referrals from generalists to specialists.” Journal of General Internal Medicine 14(5): 281-6.

Elwyn GJ, S. N. 1994. “Avoidable referrals? Analysis of 170 consecutive referrals to secondary care.” British Medical Journal 309(6954): 576-8.

Emerson, R. M., H. C. Williams, and B. R. Allen. 1998. “Severity distribution of atopic dermatitis in the community and its relationship to secondary referral.” British Journal of Dermatology 139(1): 73-76.

Eveland AP, D. G., Schafer E, Sprinkel C, Davis S, Rumpf M. 1998. “Analysis of health service areas: another piece of the psychiatric workforce puzzle.” Psychiatric Services 49(7): 956-60.

Ferris TG, C. Y., Blumenthal D, Pearson SD. 2001. “Leaving gatekeeping behind--effects of opening access to specialists for adults in a health maintenance organization.” New England Journal of Medicine 345(18): 1312-7.

Ferris TG, C. Y., Perrin JM, Blumenthal D, Pearson SD. 2002. “Effects of removing gatekeeping on specialist utilization by children in a health maintenance organization.” Archives of Pediatrics & Adolescent Medicine 156(6): 574-9.

Ferris TG, P. J., Manganello JA, Chang Y, Causino N, Blumenthal D. 2001. “Switching to gatekeeping: changes in expenditures and utilization for children.” Pediatrics 108(2): 283-90.

Forrest, C. B., P. Nutting, J. J. Werner, B. Starfield, S. von Schrader, and C. Rohde. 2003. “Managed health plan effects on the specialty referral process: results from the Ambulatory Sentinel Practice Network referral study.” Medical Care 41(2): 242-53.

Forrest, C. B. and R. J. Reid. 2001. “Prevalence of health problems and primary care physicians' specialty referral decisions.” The Journal of Family Practice 50(5): 427-32.

Forrest CB, G. G., Starfield B, Baker AE, Kang M, Reid RJ. 1999. “Gatekeeping and referral of children and adolescents to specialty care.” Pediatrics 104(1 Pt 1): 28-34.

Forrest CB, G. G., Baker AE, Bocian AB, Kang M, Starfield B. 1999. “The pediatric primary-specialty care interface: how pediatricians refer children and adolescents to specialty care.” Archives of Pediatrics & Adolescent Medicine 153 (7): 705-14.

Forrest CB, G. G., Baker AE, Bocian A, von Schrader S, Starfield B. 2000. “Coordination of specialty referrals and physician satisfaction with referral care.” Archives of Pediatrics & Adolescent Medicine 154(5): 499-506.

Forrest CB, N. P., Starfield B, von Schrader S. 2002. “Family physicians' referral decisions: results from the ASPN referral study.” The Journal of Family Practice 51(3): 215-22.

Forrest CB, N. P., von Schrader S, Rohde C, Starfield B. 2006. “Primary care physician specialty referral decision making: patient, physician, and health care system determinants.” Medical Decision Making 26(1): 76-85.

Forrest CB, R. R. 1997. “Passing the baton: HMOs' influence on referrals to specialty care.” Health Affairs (Millwood) 16(6): 157-62.

Forrest CB, S. E., Nutting PA, Starfield B. 2007. “Specialty referral completion among primary care patients: results from the ASPN Referral Study.” Annals of Family Medicine 5(4): 361-7.

Forrest CB, W. J., Fowles J, Vogeli C, Frick KD, Lemke KW, Starfield B. 2001. “Self-referral in point-of-service health plans.” Journal Of the American Medical Association 285(17): 2223-31.

Fox AT, P. R., Crossley JG, Sekaran D, Trewavas ES, Davies HA. 2004. “Improving the quality of outpatient clinic letters using the Sheffield Assessment Instrument for Letters (SAIL).” Medical Education 38(8): 852-8.

Franks, P., C. Mooney, and M. Sorbero. 2000. “Physician referral rates: style without much substance?” Medical Care

38(8): 836-46.

Franks, P., G. C. Williams, J. Zwanziger, C. Mooney, and M. Sorbero. 2000. “Why do physicians vary so widely in their referral rates?” Journal of General Internal Medicine 15(3): 163-68.

Franks P, C. C., Nutting PA. 1997. “Defining primary care. Empirical analysis of the National Ambulatory Medical Care Survey.” Medical Care 35(7): 655-68.

Franks P, C. C. 1997. “Referrals of adult patients from primary care: demographic disparities and their relationship to HMO insurance.” The Journal of Family Practice 45(1): 47-53.

Franks P, Z. J., Mooney C, Sorbero M. 1999. “Variations in primary care physician referral rates.” Health Services Research 34(1 Pt 2): 323-9.

Gerrity M, W. K. 1995. “Physicians' reactions to uncertainty: refining the constructs and scales.” Motivation and Emotion 19: 175-91.

Gerrity MS, D. R., Earp JA. 1990. “Physicians' reactions to uncertainty in patient care. A new measure and new insights.” Medical Care 28(8): 724-36.

Geyman JP, B. T., Rivers K. 1976. “Referrals in family practice: A comparative study by geographic region and practice setting.” The Journal of Family Practice 3(2): 163-7.

Glade GB, F. C., Starfield B, Baker AE, Bocian AB, Wasserman RC. 2002. “Specialty referrals made during telephone conversations with parents: a study from the pediatric research in office settings network.” Ambulatory Pediatrics 2(2): 93-8.

Glyngdal P, S. P., Kistrup K. 2002. “Non-compliance in community psychiatry: failed appointments in the referral system to psychiatric outpatient treatment.” Nordic Journal of Psychiatry 56(2): 151-6.

Gonzalez J, W. J. J., Noël PH, Lee S. 2005. “Adherence to mental health treatment in a primary care clinic.” The Journal of the American Board of Family Practice 18(2): 87-96.

Goodman DC, F. E., Bubolz TA, Mohr JE, Poage JF, Wennberg JE. 1996. “Benchmarking the US physician workforce. An alternative to needs-based or demand-based planning.” Journal Of the American Medical Association 276(22): 1811-7.

Grace JF, A. D. 1987. “Referral to hospital: perceptions of patients, general practitioners and consultants about necessity and suitability of referral.” Family Practice 4(3): 170-5.

Grembowski D, P. D., Diehr P, Katon W, Martin D, Patrick DL. 2007. “Managed care and patient ratings of the quality of specialty care among patients with pain or depressive symptoms.” BMC Health Services Research 7: 22.

Grembowski, D. E., D. Martin, D. L. Patrick, P. Diehr, W. Katon, B. Williams, R. Engelberg, L. Novak, D. Dickstein, R. Deyo, and H. I. Goldberg. 2002. “Managed care, access to mental health specialists, and outcomes among primary care patients with depressive symptoms.” Journal of General Internal Medicine 17(4): 258-69.

Grembowski DE, D. P., Novak LC, Roussel AE, Martin DP, Patrick DL, Williams B, Ulrich CM. 2000. “Measuring the "managedness" and covered benefits of health plans.” Health Services Research 35(3): 707-34.

Grembowski DE, M. D., Diehr P, Patrick DL, Williams B, Novak L, Deyo R, Katon W, Dickstein D, Engelberg R, Goldberg H. 2003. “Managed care, access to specialists, and outcomes among primary care patients with pain.” Health Services Research 38(1 Pt 1): 1-19.

Grunebaum M, L. P., Callahan M, Leon AC, Olfson M, Portera L. 1996. “Predictors of missed appointments for psychiatric consultations in a primary care clinic.” Psychiatric Services 47(8): 848-52.

Guevara JP, G. P., Shera D, Shea JA, Bauer L, Schwarz DF. 2008. “Development and psychometric assessment of the collaborative care for attention-deficit disorders scale.” Ambulatory Pediatrics 8(1): 18-24.

Haggerty JL, P. R., Beaulieu MD, Brunelle Y, Gauthier J, Goulet F, Rodrigue J. 2008. “Practice features associated with patient-reported accessibility, continuity, and coordination of primary health care.” Annals of Family Medicine 6(2): 116-23.

Harrison, R., W. Clayton, and P. Wallace. 1996. “Can telemedicine be used to improve communication between primary and secondary care?” British Medical Journal 313(7069): 1377-80.

Hartley D, K. N., Bird D, Agger M. 1998. “Management of patients with depression by rural primary care practitioners.” Archives of Internal Medicine 7(2): 139-45.

Henley, E. 1985. “An analysis of referrals and referral rates. .” Hospital & Health Services Administration 30(5): 120-9.

Hillis G, A. D. 1990. “Rejection of psychiatric treatment.” Psychiatric Bulletin 14: 149-50.

Horwitz SM, K. K., Stein RE, Storfer-Isser A, Youngstrom EA, Park ER, Heneghan AM, Jensen PS, O'Connor KG, Hoagwood KE. 2007. “Barriers to the identification and management of psychosocial issues in children and maternal depression.” Pediatrics 119(1): 208-18.

Hwang AH, H. M., Xie HW, Hardy BE, Skaggs DL. 2005. “Access to urologic care for children in California: Medicaid versus private insurance.” Urology 66(1): 170-3.

Jenkins, R. 1993. “Quality of general practitioner referrals to outpatient departments: assessment by specialists and a general practitioner.” British Journal of General Practice 43(368): 111-3.

Jiwa M, D. K., Ross J, Shaw T, Wilcox H, Spilbury K. 2009. “An inclusive approach to raising standards in general practice: working with a 'community of practice' in Western Australia.” BMC Medical Research Methodology 9: 13.

Jiwa M, M. N., Walters S. 2002. “Quality of information on referrals to colorectal surgeons: towards consensus.” Current Medical Research and Opinion 18(2): 72-7.

Jiwa M, W. S., Mathers N. 2004. “Referral letters to colorectal surgeons: the impact of peer-mediated feedback.” British Journal of General Practice 54(499): 123-6.

Johnson EK, D. S., Zhang Y, Lee CT. . 2008. “Patterns of hematuria referral to urologists: does a gender disparity exist?. .” Urology 72(3): 498-502.

Kapur K, J. G., Van Vorst KA, Escarce JJ. 2000. “Expenditures for physician services under alternative models of managed care.” Medical Care Research and Review 57(2): 161-81.

Karp, W. B., R. K. Grigsby, M. McSwiggan-Hardin, S. Pursley-Crotteau, L. N. Adams, W. Bell, M. E. Stachura, and W. P. Kanto. 2000. “Use of telemedicine for children with special health care needs.” Pediatrics 105(4 Pt 1): 843-47.

Keely E, M. K., Dojeiji S. 2002. “Can written communication skills be tested in an objective structured clinical examination format?” Academic Medicine 77(1): 82-6.

Keely E, M. K., Dojeiji S, Campbell C. 2007. “Peer assessment of outpatient consultation letters--feasibility and satisfaction.” BMC Medical Education 7: 13.

Kentish R, J. P., Lask B. 1987. “Study of written communication between general practitioners and departments of child psychiatry.” Journal of the Royal College of General Practice 37(297): 162-3.

Kern, L. M., R. Dhopeshwarkar, Y. Barron, A. Wilcox, H. Pincus, and R. Kaushal. 2009. “Measuring the effects of health information technology on quality of care: a novel set of proposed metrics for electronic quality reporting.” The Joint Commission Journal on Quality and Patient Safety 35(7): 359-69.

Killaspy H, B. S., King M, Lloyd M. 2000. “Prospective controlled study of psychiatric out-patient non-attendance. Characteristics and outcome.” British Journal of Psychiatry 176: 160-5.

Kim Y, C. A., Keith E, Yee HF Jr, Kushel MB. 2009. “Not perfect, but better: primary care providers' experiences with electronic referrals in a safety net health system.” Journal of General Internal Medicine 24(5): 614-9.

Kirchner JE, O. R., Dockter N, Kramer TL, Henderson K, Armitage T, Allee E. 2008. “Equity in veterans' mental health care: Veterans Affairs medical center clinics versus community-based outpatient clinics.” American Journal of Medical Quality 23(2): 128-35.

Knesper DJ, W. J., Pagnucco DJ. 1984. “Mental health services providers' distribution across countries in the United States.” The American Psychologist 39(12): 1424-34.

Knottnerus JA, J. J., Daams J. 1990. “Comparing the quality of referrals of general practitioners with high and average referral rates: an independent panel review.” British Journal of General Practice 40(334): 178-81.

Lambert D, A. M. 1995. “Access of rural AFDC Medicaid beneficiaries to mental health services.” Health Care Financing Review 17(1): 133-45.

Larsen DL, A. C., Hargreaves WA, Nguyen TD. 1979. “Assessment of client/patient satisfaction: development of a general scale.” Evaluation and Program Planning 2(3): 197-207.

Latinovic R, G. M., Ridsdale L. . 2006. “Headache and migraine in primary care: consultation, prescription, and referral rates in a large population. .” Journal of Neurology, Neurosurgery & Psychiatry 77(3): 385-7.

Lawler, F. H., R. S. Bisonni, and S. J. Spann. 1990. “Documentation of referrals: recording bias due to patient insurance type.” The Family Practice Research Journal 10(2): 143-50.

LeVois M, N. T., Attkisson CC. 1981. “Artifact in client satisfaction assessment: experience in community mental health settings.” Evaluation and Program Planning 4(2): 139-50.

Linn LS, Y. J., Cope D, Leake B. 1985. “Health status, job satisfaction, job stress, and life satisfaction among academic and clinical faculty.” Journal Of the American Medical Association 254(19): 2775-82.

Ludke, R. 1982. “An examination of the factors that influence patient referral decisions.” Medical Care 20(8): 782-96.

Lungen M, S. B., Messner P, Lauterbach KW, Gerber A. 2008. “Waiting times for elective treatments according to insurance status: A randomized empirical study in Germany.” International Journal for Equity in Health 9(7): 1-7.

Massman NJ, D. J., Fortman KK, Schwartz KJ, Solem LD. 1999. “Burns follow-up: an innovative application of telemedicine.” Journal of Telemedicine and Telecare 5(Suppl 1): S52-4.

Matas M, S. D., Griffin W. 1992. “A profile of the noncompliant patient: a thirty-month review of outpatient psychiatry referrals.” General Hospital Psychiatry 14(2): 124-30.

Mayer, M. 2008. “Disparities in geographic access to pediatric subspecialty care.” Maternal and Child Health Journal 12(5): 624-32.

McPhee SJ, L. B., Saika GY, Meltzer R. 1984. “How good is communication between primary care physicians and subspecialty consultants?” Archives of Internal Medicine 144(6): 1265-8.

Mead N, B. P., Roland M. 2008. “The General Practice Assessment Questionnaire (GPAQ) - development and psychometric characteristics.” BMC Family Practice 9: 13.

Menken M, B. R., Lee P. 1990. “Neurology referral patterns.” HMO Practice 4(2): 57-60.

Metcalfe DHH, S. D. 1974. “Patterns of referral from family practices.” The Journal of Family Practice 1(2): 34-38.

Mojtabai, R. 2005. “Compliance with mental health and other specialty care referrals among Medicare/Medicaid dual enrollees.” Community Mental Health Journal 41(3): 339-44.

Mommsen S, A. J., Sell A. 1983. “Presenting symptoms, treatment delay and survival in bladder cancer.” Scandinavian Journal of Urology and Nephrology 17(2): 163-7.

Moore AT, R. M. 1989. “How much variation in referral rates among general practitioners is due to chance?” British Medical Journal 298(6672): 500-2.

Moscovice I, S. C., Shortell SM. 1979. “Referral patterns of family physicians in an underserved rural area.” The Journal of Family Practice 9(4): 677-82.

Mulhausen R, M. J. 1989. “Physician need. An alternative projection from a study of large, prepaid group practices.” Journal Of the American Medical Association 261(13): 1930-4.

Myers K, K. E., Dojeiji S. 1999. “Evaluating the communication skills of residents: development of a rating scale to evaluate consultation letters.” Academic Medicine 74 suppl: S111-S13.

Myrdal G, L. M., Hillerdal G, Lamberg K, Agustsson T, Ståhle E. 2004. “Effect of delays on prognosis in patients with non-small cell lung cancer.” Thorax 59(1): 45-9.

Newhouse JP, W. A., Bennett BW, Schwartz WB. 1982. “Where have all the doctors gone?” Journal Of the American Medical Association 247(17): 2392-6.

Newton, J., A. Hutchinson, V. Hayes, E. McColl, I. Mackee, and C. Holland. 1994. “Do clinicians tell each other enough? An analysis of referral communications in two specialties.” Family Practice 11(1): 15-20.

Nguyen LT, M. N., Franzen BJ, Ahrenholz DH, Sorensen NW, Mohr WJ 3rd, Solem LD. 2004. “Telemedicine follow-up of burns: lessons learned from the first thousand visits.” Journal of Burn Care & Rehabilitation 25(6): 485-90.

Niefeld MR, K. J. 2005. “Access to ambulatory medical and long-term care services among elderly Medicare and Medicaid beneficiaries: organizational, financial, and geographic barriers.” Medical Care Research and Review 62(3): 300-19.

Noone A, G. M., Coulter A, Seagroatt V. 1989. “Do referral rates vary widely between practices and does supply of services affect demand? A study in Milton Keynes and the Oxford region.” Journal of the Royal College of General Practice 39(327): 404-7.

Nosarti, C., T. Crayford, J. V. Roberts, E. Elias, K. McKenzie, and A. S. David. 2000. “Delay in presentation of symptomatic referrals to a breast clinic: patient and system factors.” British Journal of Cancer 82(3): 742-48.

Nyman, J. A., W. G. Manning, S. Samuels, and B. F. Morrey. 1998. “Can specialists reduce costs? The case of referrals to orthopaedic surgeons.” Clinical Orthopaedics and Related Research 48(350): 257-67.

Oiesvold T, S. M., Hansson L, Christiansen L, Göstas G, Lindhardt A, Saarento O, Sytema S, Zandrén T. 1998. “Factors associated with referral to psychiatric care by general practitioners compared with self-referrals.” Psychological Medicine 28(2): 427-36.

Olfson, M. 1991. “Primary care patients who refuse specialized mental health services.” Archives of Internal Medicine 151(1): 129-32.

O'Malley AS, C. P. 2009. “Patient experiences with coordination of care: the benefit of continuity and primary care physician as referral source.” Journal of General Internal Medicine 24(2): 170-7.

Otters H, v. d. W. J., Schellevis FG, van Suijlekom-Smit LW, Koes BW. 2004. “Dutch general practitioners' referral of children to specialists: a comparison between 1987 and 2001.” British Journal of General Practice 54(508): 848-52.

Parchman ML, N. P., Lee S. 2005. “Primary care attributes, health care system hassles, and chronic illness.” Medical Care 43(11): 1123-9.

Parker, G., M. Wright, S. Robertson, and A. Sengoz. 1996. “To whom do you refer? A referrer satisfaction study.” Australian and New Zealand Journal of Psychiatry 30(3): 337-42.

Parkerson GR Jr, B. W., Tse CK. 1993. “The Duke Severity of Illness Checklist (DUSOI) for measurement of severity and comorbidity.” Journal of Clinical Epidemiology 46(4): 379-93.

Parloff MB, K. H., Frank JD. 1954. “Comfort, effectiveness, and self-awareness as criteria of improvement in psychotherapy.” The American Journal of Psychiatry 111(5): 343-52.

Parsons RJ, B. K. 1998. “Rural referrals represent significant revenue for managed care organizations.” Healthcare Financial Management 52(9): 43-6.

Pearson SD, G. L., Orav EJ, Guadagnoli E, Garcia TB, Johnson PA, Lee TH. 1995. “Triage decisions for emergency department patients with chest pain: do physicians' risk attitudes make the difference?” Journal of General Internal Medicine 10(10): 557-64.

Peeters FP, B. H. 1999. “'No-show' for initial screening at a community mental health centre: rate, reasons and further help-seeking.” Social Psychiatry and Psychiatric Epidemiology 34(6): 323-7.

Peterson, B. D., H. A. Pincus, A. Suarez, and D. A. Zarin. 1998. “Referrals to psychiatrists.” Psychiatric Services 49(4): 449.

Pincus, H. A., B. D. Peterson, A. P. Suarez, and D. A. Zarin. 1998. “The structure of psychiatrists' outpatient practice.” Psychiatric Services 49(6): 747.

Prasher V P, F.-m. D., Krishnan V H R, Oycbode F. 1992. “Communication between general practitioners and psychiatrics.” Psychiatric Bulletin 16: 468-9.

Prentice JC, P. S. 2007. “Delayed access to health care and mortality.” Health Services Research 42(2): 644-62.

Pullen IM, Y. A. 1985. “Is communication improving between general practitioners and psychiatrists?” British Medical Journal 290(6461): 31-3.

Redfern J, B. A. 2000. “Efficiency of care at the primary-secondary interface: variations with GP fundholding.” Health Place 6(1): 15-23.

Redlick F, R. B., Gomez M, Fish JS. 2002. “An initial experience with telemedicine in follow-up burn care.” Journal of Burn Care & Rehabilitation 23(2): 110-5.

Reschovsky JD, O. M. A. 2008. “Do primary care physicians treating minority patients report problems delivering high-quality care?. .” Health Affairs 27(3): 222-31.

Reschovsky JD, S. A. 2005. “Access and quality: does rural America lag behind?” Health Affairs (Millwood) 24(4): 1128-39.

Reynolds GA, C. J., Roland MO. 1991. “General practitioner outpatient referrals: do good doctors refer more patients to hospital?” British Medical Journal 302(6787): 1250-2.

Rhodes KV, V. T., Kushner H, Levy H, Asplin BR. 2009. “Referral without access: for psychiatric services, wait for the beep.” Annals of Emergency Medicine 54(2): 272-8.

Rickels K, G. C., Lipman RS, Derogatis LR, Fisher EL. 1976. “The Hopkins Symptom Checklist. Assessing emotional distress in obstetric-gynecologic practice.” Primary Care 3(4): 751-64.

Rodriguez HP, v. G. T., Rogers WH, Safran DG. 2009. “Organizational and market influences on physician performance on patient experience measures.” Health Services Research 44(3): 880-901.

Roland, M., J. Grimshaw, R. Grol, D. Shanks, A. Johnson, I. Russell, and R. Taylor. 1997. “Do general practitioner attitudes and characteristics of their practices explain patterns of specialist referral?” The European Journal of General Practice 3(143-7).

Roland, M. and R. Morris. 1988. “Are Referrals by General Practitioners Influenced by the Availability of Consultants?” British Medical Journal 297: 599-600.

Roland, M. O., R. W. Porter, J. G. Matthews, J. F. Redden, G. W. Simonds, and B. Bewley. 1991. “Improving Care: A Study of Orthopaedic Outpatient Referrals.” British Medical Journal 302: 1124-28.

Roland MO, B. J., Morrell DC, McDermott A, Paul E. 1990. “Understanding hospital referral rates: a user's guide.” British Medical Journal 301(6743): 98-102.

Rosenthal MB, Z. A., Newhouse JP. 2005. “The geographic distribution of physicians revisited.” Health Services Research 40(6 Pt 1): 1931-52.

Rostand SG, K. K., Rutsky EA, Pate BA. 1982. “Racial differences in the incidence of treatment for end-stage renal disease.” New England Journal of Medicine 306(21): 1276-9.

Ruane, T. J. 1979. “Consultation and Referral in a Vermont Family Practice: A Study of Utilization, Specialty Distribution, and Outcome.” The Journal of Family Practice 5: 1037-40.

Rubin HR, G. B., Rogers WH, Kosinski M, McHorney CA, Ware JE Jr. 1993. “Patients' ratings of outpatient visits in different practice settings. Results from the Medical Outcomes Study.” Journal Of the American Medical Association 270(7): 835-40.

Salem-Schatz S, M. G., Rucker M, Pearson SD. 1994. “The case for case-mix adjustment in practice profiling. When good apples look bad.” Journal Of the American Medical Association 272(11): 871-4.

Salomaa ER, S. S., Hiekkanen H, Liippo K. 2005. “Delays in the diagnosis and treatment of lung cancer.” Chest 128(4): 2282-8.

Scheffler R, I. S. 1998. “Mental health staffing in managed care organizations: a case study.” Psychiatric Services 49(10): 1303-8.

Schoenman, J. A., W. N. Evans, and C. L. Schur. 1997. “Primary care case management for Medicaid recipients: evaluation of the Maryland Access to Care program.” Inquiry 34(2): 155-70.

Schwartz WB, N. J., Bennett BW, Williams AP. 1980. “The changing geographic distribution of board-certified physicians.” New England Journal of Medicine 303(18): 1032-8.

Scott B, B. R., Lohr KN, Goldberg GA. 1981. “Conceptualization and measurement of physiologic health for adults. Vol 10. Joint disorders.”. Santa Monica, California: The RAND Corporation.

Shea D, S. B., Vasey J, Nag S. 1999. “Medicare physician referral patterns.” Health Services Research 34(1 Pt 2): 331-48.

Shi L, S. B., Xu J. 2001. “Validating the adult primary care assessment tool.” The Journal of Family Practice 50(2): 161-71.

Shi L, S. B., Xu J, Politzer R, Regan J. 2003. “Primary care quality: community health center and health maintenance organization.” Southern Medical Journal 96(8): 787-95.

Shortell SM, V. S. 1975. “Patient referral differences among specialties.” Health Services Research 10(2): 146-61.

Sicras-Mainar A, S.-T. J., Navarro-Artieda R, Llausí-Sellés R, Ruano-Ruano I, González-Ares JA. 2007. “Adjusted Clinical Groups use as a measure of the referrals efficiency from primary care to specialized in Spain.” European Journal of Public Health 17(6): 657-63.

Simunovic M, G. A., McCready D, Coates A, Levine M, DePetrillo D. 2001. “A snapshot of waiting times for cancer surgery provided by surgeons affiliated with regional cancer centres in Ontario.” Canadian Medical Association Journal 165(4): 421-5.

Skaggs DL, C. S., Vitale MG, Femino JD, Kay RM. 2001. “Access to orthopedic care for children with medicaid versus private insurance in California.” Pediatrics 107(6): 1405-8.

Smyth, F., J. Owens, and T. Carey. 1994. “Communication between GPs and psychiatrists.” Irish Medical Journal 87(3): 88-89.

Spurgeon P, B. F., Kerr D. 2000. “Waiting times for cancer patients in England after general practitioners’ referrals: retrospective national survey.” British Medical Journal 320(7238): 838-9.

Starfield B, C. C., Nanda J, Forrest CB, Berk R. 1998. “Consumer experiences and provider perceptions of the quality of primary care: implications for managed care.” The Journal of Family Practice 46(3): 216-26.

Starfield B, F. C., Nutting PA, von Schrader S. 2002. “Variability in physician referral decisions.” The Journal of the American Board of Family Practice 15(6): 473-80.

Stille CJ, K. N., Primack WA. 2003. “Generalist-subspecialist communication about children with chronic conditions: an analysis of physician focus groups.” Ambulatory Pediatrics 3(3): 147-53.

Stille CJ, M. T., Primack WA, Mazor KM, Wasserman RC. 2006. “Determinants and impact of generalist-specialist communication about pediatric outpatient referrals.” Pediatrics 118(4): 1341-9.

Stille CJ, P. W., Savageau JA. 2003. “Generalist-subspecialist communication for children with chronic conditions: a regional physician survey.” Pediatrics 112(6 Pt 1): 1314-20.

Stille CJ, P. W., McLaughlin TJ, Wasserman RC. 2007. “Parents as information intermediaries between primary care and specialty physicians.” Pediatrics 120(6): 1238-46.

Sturm R, J. C., Meredith LS, Yip W, Manning WG, Rogers WH, Wells KB. 1995. “Mental health care utilization in prepaid and fee-for-service plans among depressed patients in the Medical Outcomes Study.” Health Services Research 30(2): 319-40.

Stuve P, B. P., Hartig P. 1989. “Trends in the rural community mental health work force: a case study.” Hospital & Community Psychiatry 40(9): 932-6.

Surender, R., J. Bradlow, A. Coulter, H. Doll, and S. S. Brown. 1995. “Prospective study of trends in referral patterns in fundholding and non-fundholding practices in the Oxford region, 1990-4.” British Medical Journal 311(7014): 1205-08.

Tabenkin H, O. B., Steinmetz D, Tamir A, Kitai E. 1998. “Referrals of patients by family physicians to consultants: a survey of the Israeli Family Practice Research Network.” Family Practice 15(2): 158-64.

Tanielian, T. L., H. A. Pincus, A. J. Dietrich, J. W. Williams, T. E. Oxman, P. Nutting, and S. C. Marcus. 2000. “Referrals to psychiatrists. Assessing the communication interface between psychiatry and primary care.” Psychosomatics 41(3): 245-52.

Trude S, S. J. 2003. “Referral gridlock: primary care physicians and mental health services.” Journal of General Internal Medicine 18(6): 442-9.

Tsang MW, R. J. J. 2006. “Even patients with changing moles face long dermatology appointment wait-times: a study of simulated patient calls to dermatologists.” Journal of the American Academy of Dermatology 55(1): 54-8.

Unit), R. C. o. G. P. B. R. 1978. “Practice activity analysis. 5. Referral to specialists.” Journal of the Royal College of General Practice 28(251-2).

van Suijlekom-Smit LW, B. M., van der Wouden JC, van der Velden J, Visser HK, Dokter HJ. 1997. “Children referred for specialist care: a nationwide study in Dutch general practice.” British Journal of General Practice 47(414): 19-23.

Vardy DA, F. T., Sherf M, Spilberg O, Goldfarb D, Cohen AD, Mor-Yosef S, Shvartzman P. 2008. “A co-payment for consultant services: primary care physicians' referral actualization.” Journal of Medical Systems 32(1): 37-41.

Von Korff M, O. J., Keefe FJ, Dworkin SF. 1992. “Grading the severity of chronic pain.” Pain 50(2): 133-49.

Ware JE Jr, H. R. 1988. “Methods for measuring patient satisfaction with specific medical encounters.” Medical Care 1988(26): 4.

Weinerman B, d. D. J., Hughes A, Robertson S. 2005. “Can subspecialty cancer consultations be delivered to communities using modern technology?--A pilot study.” Telemed and e-Health. 11(5): 608-15.

West J, K. J., Pion GM, et al. . 2001. “Mental health practitioners and trainees. In:Mandercheid RW, Henderson MJ, eds. Mental Health, United States, 2000.”. Washington, DC: Superintendent of Documents, US Government Printing Office; 2001.

Westerman RF, H. F., Bezemer PD, Gort G. 1990. “A study of communication between general practitioners and specialists.” British Journal of General Practice 40(340): 445-9.

Wiles, C. M. and M. Lindsay. 1996. “General practice referrals to a department of neurology.” The Journal of the Royal College of Physicians of London 30(5): 426-31.

Wilkin, D. and A. G. Smith. 1987. “Variation in General Practitioners' Referral Rates to Consultants.” Journal of the Royal College of General Practice 37: 350-53.

Williams AP, S. W., Newhouse JP, Bennett BW. 1983. “How many miles to the doctor?” New England Journal of Medicine 309(16): 958-63.

Williams, P. T. and G. Peet. 1994. “Differences in the value of clinical information: referring physicians versus consulting specialists [see comments].” The Journal of the American Board of Family Practice 7(4): 292-302.

Young DW, P. J., Davis WA, Harman D, Williams RS. 1985. “Out-patient letters: requirement and contents.” Effective Health Care 2(6): 225-9.

Zarin DA, P. H., Peterson BD, West JC, Suarez AP, Marcus SC, McIntyre JS. 1998. “Characterizing psychiatry with findings from the 1996 National Survey of Psychiatric Practice.” The American Journal of Psychiatry 155(3): 397-404.
